# Supplementary material for: Characteristics of COVID-19 Inpatients in Rehabilitation Units during the First Pandemic Wave: A Cohort Study from a Large Hospital in Champagne Region
Source: Biology (Basel). 2022 Jun 20;11(6):937. doi: 10.3390/biology11060937 (PMC9219626; doi:10.3390/biology11060937)
Supplement: Supplementary file 1 [file biology-11-00937-s001.zip › biology-1750678-supplementary.pdf]

Supplementary Table S1 : Characteristics of patients according to discharge destination : Home versus post acute care unit

**Characteristic**	**Home N=233**		**PRM + PRU N=111**		***p value***
Women *(n, %)*	120	(51.5%)	50	(45%)	*0.263*
Age, years *(median, [Q1-Q3])*	67	[53-79]	76	[69-84]	*< 0.001*
BMI Kg/m² ‡ *(median, [Q1-Q3])*	29	[25-32]	26	[23-30]	*0.002*
BMI ≥ 30 Kg/m² ‡ *(n, %)*	13	(5.6%)	4	(3.6%)	*0.298*
Current smokers * (n, %)	14	(6.0%)	4	(3.6%)	*0.267*
Arterial hypertension (n, %)	117	(50.2%)	67	(60.4%)	*0.553*
Diabetes (n, %)	54	(23.2%)	32	(28.8%)	*0.630*
Chronic respiratory disease (n, %)	40	(17.2%)	24	(21.6%)	*0.652*
Renal failure requiring RRT (n, %)	14	(6.0%)	7	(6.3%)	*0.859*
Charlson score (médiane, [Q1-Q3])	1	[0-2]	2	[0-3]	*0.055*
At risk of severe form of disease (n, %)	194	(83.3%)	105	(94.6%)	*0.004*

PRM = Physical and Rehabilitation; PRU = polyvalent rehabilitation unit ; BMI = body mass index ; Q, quartile (Q1 = 25th percentile, Q3 = 75th percentile) ; RRT, renal replacement therapy.
‡ 82 missing data, * 40 missing data.

Supplementary Table S2: Clinical presentation, biology results and treatment of patients according to discharge destination : home versus post acute care unit

	**Home (A) N=233**		**PRM + PRU N=111**		***p value***
Clinical presentation					
Severe clinical presentation *(n, %)*	60	(25.8%)	35	(31.5%)	*0.262*
Early warning score † *(median, [Q1-Q3])*	6	[3-8]	7	[4-9]	*0.008*
Oxygen therapy at admission *(n, %)*	89	(38.2%)	55	(49.6%)	*0.035*
Breathing rate *(median, [Q1-Q3])*	22	[18-26]	20	[18-28]	*0.764*
Pulmonary embolism *(n, %)*	10	(4.3%)	7	(6.3%)	*0.436*
Biology during acute infection					
Leukocytes, G/L *(median, [Q1-Q3])*	6.9	[4.9-9]	6.1	[4.2-8.9]	*0.192*
Lymphocytes <1.5 G/L *(n, %)*	179	(76.8%)	88	(79.3%)	*0.196*
GFR < 60 ml/min/kg *(n, %)*	56	(24%)	42	(37.8%)	*0.009*
Albuminemia, g/L Ŧ *(median, [Q1-Q3])*	35	[31-38]	32	[29-36]	*0.004*
CRP, mg/L *(median, [Q1-Q3])*	61,6	[22.5-120]	67	[20.5-146]	*0.752*
Troponin, ng/L ¥ *(median, [Q1-Q3])*	13,8	[6.2-34.8]	21.8	[14.2-37.7]	*< 0.001*
Treatments					
Need for ICU admission *(n, %)*	29	(12.4%)	36	(32.4%)	*< 0.001*
Antibiotic therapy *(n, %)*	217	(93.1%)	107	(96.4%)	*0.227*
Corticosteroid therapy *(n, %)*	122	(52.4%)	68	(61.3%)	*0.131*
Anticoagulant therapy *(n, %)*	219	(94.0%)	107	(96.4%)	*0.349*
Length of stay, days *(median, [Q1-Q3])*	8	[5-14]	16	[10-29,5]	*< 0.001*

PRM = Physical and Rehabilitation ; PRU = polyvalent rehabilitation unit ; Q, quartile (Q1 = 25th percentile, Q3 = 75th percentile) ; GFR, glomerular filtration rate ; CRP, C-reactive protein ; ICU, intensive care unit.

† 35 missing data, Ŧ 39 missing data, ¥ 75 not applicable
